# Early penile prosthesis implantation in refractory ischaemic priapism: a narrative review

**DOI:** 10.1038/s41443-025-01199-z

**Published:** 2025-11-05

**Authors:** Ross Calopedos, Davide Trubbia, David Ralph, Wai Gin Lee

**Affiliations:** 1 East Sydney Private Hospital, Sydney, Australia; 2 Department of Urology, University College London Hospitals, London, UK; 3 Division of Surgery and Interventional Sciences, University College London, London, UK

**Keywords:** Surgery, Sexual dysfunction

## Abstract

Ischaemic priapism (IP) is a urological emergency that can lead to permanent erectile dysfunction (ED) and penile deformity if not promptly managed. Refractory ischaemic priapism (RIP), defined as IP persisting beyond 48 hours despite conventional interventions, necessitates a paradigm shift in treatment strategy. Early penile prosthesis (PP) implantation offers a proactive approach in surgical management to preserve penile function while minimising complications associated with delayed surgery. This review synthesises current evidence on early PP insertion for RIP, evaluating its advantages over delayed intervention. We examine the pathophysiological progression of IP, emphasising corporal smooth muscle necrosis and penile fibrosis, which render erectile function recovery unlikely even with successful detumescence. Limitations of shunting procedures are also discussed and emerging diagnostic tools highlighted, such as magnetic resonance imaging (MRI), for assessing irreversible corporal damage. Comparative analysis of early versus delayed PP insertion reveals superior patient satisfaction, lower revision rates, reduced penile shortening, and fewer perioperative complications with early implantation. Device selection, operative techniques, and risk mitigation strategies are also explored. Our findings support early PP implantation as the preferred management strategy for RIP, aligning with international guidelines. Further research is warranted to refine surgical protocols and optimise patient outcomes.

## Introduction

Ischemic priapism is a urological emergency defined by erection lasting more than 4 hours in the absence of sexual stimulation or despite orgasm [1]. Ischaemic priapism (IP) makes up about 95% of cases and is characterised by a painful, self-perpetuating compartment syndrome of the corpus cavernosa [1]. Impaired arterial inflow results in time-dependent corporal smooth muscle (CSM) damage, which can result in permanent erectile dysfunction (ED) and penile deformity (loss of length/girth and penile curvature) if not reversed promptly [1]

Early penile prosthesis (PP) implantation for refractory ischaemic priapism (RIP) represents a paradigm shift in emergency management of this condition [2]. We define RIP as IP > 48 h that has not responded to conservative management (aspiration, corporal washout, intracavernosal sympathomimetics) [1]. Traditionally, a stepwise algorithm of increasing invasiveness has been applied to reverse IP, irrespective of time course [2]. However, ED is inevitable in RIP despite successful detumescence, and subsequent PP can be complicated by prior shunting or by corporal fibrosis if PP is delayed too long [1].

There is still much deliberation about the precise timing, type of PP or specific technique best employed in each situation. In this article, we present a narrative review of the current literature with the aim of supporting management decisions with men presenting with RIP.

## Pathophysiology

Progressive hypoxia, hypercapnia, glucopenia and acidosis result in pain and oxidative stress [3]. CSM relaxation promotes priapic episodes and is maintained by disruptions in normal endothelial signalling pathways and as a direct effect of excess reactive oxygen species [4, 5]. Hypoxia also independently up-regulates key enzymes in the adenosine pathway, thereby increasing free adenosine which causes CSM relaxation [6]. Unremitting compartment syndrome results in ischaemia-related cell death, through activation of apoptotic pathways and necrosis via hypoxia-inducible factor (HIF-1) [7]. This process sheds light on failure of sympathomimetics to resolve IP in refractory cases, as they rely on contraction of viable CSM tissue [3]. Progressive sinusoidal tissue damage leads to a pro-inflammatory cascade, characterised by increased Transforming Growth Factor-beta (TGF-β) expression [4]. TGF-β results in fibroblast proliferation, activation of matrix metalloproteinases (MMP) and the inexorable deposition of collagen [4]. In this way, permanent functional and morphological changes arise.

## To Shunt or not to Shunt?

Shunting may achieve detumescence during RIP after failed first line management (aspiration, corporal washout, intracavernosal sympathomimetics) [1]; however preservation of erectile function is unlikely [8].

If detumescence is achieved within 24 hours of IP onset, about 50% of patients are expected to recover erectile function [8]. Despite successful shunting, if IP lasts 24-36 hours 75% developed moderate-severe ED (5 item International Index of Erectile Function IIEF-5 < 17). After 48 hours, all 28 men of one series developed severe ED and underwent PP implantation [8]. This trend is corroborated by results of other series [9–11] as well as histopathological changes observed over time. Interstitial oedema and the beginnings of cavernous endothelial destruction are observed at 12 hours following IP onset. As endothelial cells slough away and ulcerate, thrombocyte adherence to the unroofed basement membrane occurs at around 24 hours. At 48 h, diffuse sinusoidal thrombosis, CSM cell necrosis and fibroblastic transformation are evident [8, 12].

Shunt procedures increase the complication rate of subsequent early PP insertion.

Weakened tunica increases the risk of distal perforation and erosion and preceding instrumentation increases the chance of infective complications. In cases where CSM dysfunction may be reversible, glans-preserving shunt techniques, such as peno-scrotal decompression, should be also considered [13]. However, where irreversible CSM changes have occurred, early PP without shunting is favourable.

## Diagnostics

Ability to identify irreversible CSM changes is key in decision making of RIP management. Based on a prospective series of 38 men with RIP, MRI had 100% sensitivity in demonstrating CSM necrosis or fibrosis [14]. If CSM necrosis is demonstrated, European Association of Urology (EAU) guidelines recommend considering early PP implantation instead of shunting. However, if shunting is performed, tissue should be obtained at this time [15]. This is mirrored by the British Association of Urological Surgeons (BAUS) guidelines that advocate early PP if MRI or penile doppler shows non-perfusion at 48-72 hours [16]. Similarly, consensus opinion of American Urological Association (AUA) / Sexual Medicine Society of North America (SMSNA) state that men who present with IP > 36 hours or who have failed distal shunting attempts, may be considered for early PP [17].

## Methods

### Data collation

In the present study, results of published articles are compiled to evaluate differences in outcomes between early and delayed PP implantation following IP, in addition to outcomes specific to device type during early implantation. Articles included in the presented tables are those that focused on PP insertion for RIP, were published in English, and reported specific outcomes and events, rather than feasibility alone. Methodology and measured variables of are heterogenous however specific event rates were compiled using available data (Tables 1–4).

**Table 1:** Summary of studies of early PP insertion for RIP.

Study	Men (n)	Mean age (yrs)	SCD (n)	Median IP Duration (range)	Initial IP Mx	Median time from IP Onset to PP (range)	Approach / Technique	Median follow-up (month / range)	Type	Intra-op Cx (n): Mx	Post-op Cx (n)	Successful Insertion (n) / Revision (n)	Penile Dimensions / Complaint of penile shortening (n)	Post-op sexual intercourse (n) / Satisfaction (n)	Elective exchange (MPP → IPP)
Rees 2002 [26]	8	41	1	91 h	CMx 8 DCS 2 CSS 3	(1-8 days)	Penoscrotal	17	MPP 6	0	0	Success (6)	NR NR	5 5	0
									IPP 2	0	1 Deformity (2° delayed activation @ 6 weeks): Revised	Success (2), Revision (1)	NR NR	2 2	
Ralph 2009 [27]	50	46	5	209 h	CMx 50 DCS 14 CSS 1	209 h (24 – 720)	Penoscrotal	15.6 (4 -60)	MPP 43	0	Infection (3), impending distal erosion (3), cylinders too short (2)	Success (43) Revision (8)	NR 0	42 48	6
									IPP 7	0	1 Deformity (2° delayed activation @ 6 weeks): Reg. cycling. 1 Auto-inflation	Success (7)			
Salem 2010 [28]	12	43	1	NR	CMx 12 DCS 11 CSS 1	125 h	Penoscrotal + Cylinder fixation	24	MPP 12	Proximal corporal perf (1): sling suture	0	Success (12)	NR NR	12 NR	0
Sedigh 2011 [19]	5	52	NR	41 h (28-56)	CMx 5	NR	Penoscrotal + IPP cylinders over-sized (0.5-1 cm)	NR	MPP 1	0	0	Success (1)	NR NR	5 5	0
									IPP 4	0	4 Reduced penile sensation, recovered in 3 months	Success (4)			
Zacharakis 2014 [20]	68	42	NR	NR	CMx 68 DCS 28	7 days (1-17)	Penoscrotal / dilatation not performed to corporal tip if DCS prior	17	MPP 64	0	5 infections (all had DCS prior) 2 distal erosions (associated with infection)	Success (68) Revision (6)	NR 2/6 (patients who needed delayed re-implant after explant)	NR 65	
									IPP 4	0	1 deformity (2° delayed IPP activation @ 3 months)				
Tausch 2015 [29]	14	NR	4	NR	NR	82 h	NR	NR	MPP 14	0	1 Infection 1 Urethral erosion 1 Distal erosion	Success (14) Revision (3)	NR NR	NR NR	0
Zacharakis 2015 [30]	10	41	2	NR	CMx 10 DCS 5	188 h (98-336)	Penoscrotal	13.5 (3-24)	MPP 10	0	0	Success (10)	NR NR	NR 8	10 (All electively exchanged for IPP median 130 days after MPP: Satisfaction increase to 90%)
Salman 2022 [21]	23	55	2	NR	CMx 15 DCS 8	5 days	Penoscrotal	11	MPP 23	Distal corporal perf (1): suture fixation	Distal erosion (2) Prosthesis infection (2): Same patients who had erosion, both had DCS Wound infection (6)	Success (23) Revision (2)	NR NR (Mean implant length 22 cm: 2 cm longer than delayed group)	NR 21 (2 patients with erosion not satisfied)	0
Barham 2023 [22]	15	NR	NR	60 h (5-144)	CMx 15 DCS 10	2 m (3 d – 6 m)	NR	3 (2-17)	IPP 15	0	0	Success (15)	NR NR	NR NR	-

*PP Penile prosthesis, *IP* Ischaemic priapism, *RIP* Refractory Ischaemic Priapism, *MPP* malleable penile prosthesis, *IPP* inflatable penile prosthesis; outcomes are subdivided by prosthesis type if specified by authors. *Cx* Complications, *CMx* Conservative management by aspiration, irrigation and intracavernosal injection of phenylephrine, *DCS* Distal corpospongiosal shunt, *CSS* Corpora-saphenous shunt. *NR* Not recorded.

**Table 2:** Summary of studies of delayed PP insertion for RIP.

Study	Men (n)	Mean age (yrs)	SCD (n)	Median IP Duration (range)	Initial IP Mx	Median time from initial IP episode to PP (range)	Approach / Technique	Adjunct Manoeuvres	Median follow-up (month)	Type	Intra-op Cx (n): Mx	Post-op Cx (n)	Successful Insertion (n) / Revision (n)	Penile Dimensions / Complaint of shortening (n)	Post-op sexual intercourse (n) / Satisfaction (n)
Bertram 1985 [23]	6	NR	NR	NR	NR	NR	NR	NR	NR	MPP 4 IPP 1	Inability (1)	NR	5 NR	NR NR	NR NR
Douglas 1990 [24]	5	22	5	NR	NR	NR*	Perineal-scrotal (4) Dorsal (1)	Sharp dissection, excavation	NR	MPP 5	Urethral injury (1)	Distal erosion (5) Urethral erosion (1)	5 5	NR NR	4 NR
Durazi 2008 [25]	17	22	16	NR	CMx 17 DCS 17 CSS 5	11 m	Ventral penile for MPP, Penoscrotal for Ambicor, Infra-pubic for 3-piece IPP	Extended corporotomy, sharp dissection, open excavation, corporal release incisions to close tunica	23	MPP 11 IPP 6	Urethral injury (2) Partial corporectomy and single cylinder insertion (1)	0	17 0	NR 5	16 NR
Sedigh 2011 [19]	3	NR	NR	NR	CMx 3 DCS 3 CCS 1	9-12 m	NR	NR	NR	IPP 3	Urethral injury (1)	0	3 0	NR NR	NR NR
Zacharakis 2014 [20]	27	45	NR	NR	NR	5 m (2-14)	Degloving 80% second distal corporotomy	Rossello dilators, 80% required downsized cylinders (narrow base Titan Coloplast® or CXR AMS®)	21	MPP 12 IPP 15	0	Infection (5) Distal erosion (1) Mechanical failure (1)	27 7	NR 11	25 16
Salman 2022 [21]	19	54	4	NR	CMx 19 DCS 13	177 days	Penoscrotal	Prolene mesh used to augment distal corpora in 4 cases	15	MPP 19	Urethral injury (4) Distal corporal perf (7) Prox corporal perf (4)	0	19 0	NR NR	19 NR
Berham 2023 [22]	47	NR	NR	33 h (3-168)	DCS 18	31.5 m (7m-23yr)	NR	NR	7 (3-24)	IPP 47	Urethral injury (1) Prox corporal perf (1)	Infection (4) Erosion (6) (corporotomy 3, prox migration 2, distal 1) Not cycling (4) Cylinder leak (2)	47 16	NR NR	NR NR

*PP Penile prosthesis, *MPP* malleable penile prosthesis, *IPP* inflatable penile prosthesis; outcomes are subdivided by prosthesis type if specified by authors. *CMx* Conservative management by aspiration, irrigation and intracavernosal injection of phenylephrine, *DCS* Distal corpospongiosal shunt, CSS = Corpora-saphenous shunt. *NR* Not recorded.

**Table 3:** Compiled outcomes of early vs delayed PP implantation for RIP.

Event/Outcomes	Early Implantation (%)	Delayed Implantation (%)
Intra-op complication rate (overall)	1 (19 –22,26-30)	18.5 (19-25)
Unable to Implant	0	1 (23)
Corporal Perforation	1 (21,28)	9.7 (21,22)
Urethral Injury	0	7.3 (21-25)
Post-op complication rate (overall)	11.2 (19 –22,26-30)	23.4 (19-25)
Infected Implant	5.4 (20,21,27,29)	7.6 (20,22)
Corporal Erosion^a^	4.4 (20,21,27,29)	11 (20,22,24)
Revision Rate^b^	9.8 (20,21,26,27,29)	23.7 (20,22,24)
Satisfaction Rate	93.9 (19,20,21,26,27,30)	60 (20)
Penile Shortening	1.7 (20, 27)	36.4 (20,25)

^a^*Corporal erosion*= any process where the cylinder moves outside the corporal space e.g. urethral erosion, impending extrusion, proximal migration etc.

^b^Excluding elective exchange of prosthesis.

**Table 4:** Compiled outcomes of early MPP vs IPP implantation for RIP.

Event / Outcome	MPP, n = 173 (%) (19-21,26-30)	IPP, n = 32 (%) (19,20,22,26,27)
**Intra-op complications:**		
Corporal Perforation	1.2 (21,28)	0
**Post-op complications:**		
Deformity	0	9.4 (20,26,27)
Infected Implant	6.4 (20,21,27,29)	0
Corporal Erosion^a^	5.2 (20,21,27,29)	0
**Revision Rate**^b^	11 (20,21,27,29)	3.1 (26)

*MPP* malleable penile prosthesis, *IPP* inflatable penile prosthesis

^a^*Corporal erosion*= any process where the cylinder moves outside the corporal space e.g. urethral erosion, impending extrusion, proximal migration etc.

^b^Excluding elective exchange of prosthesis.

A comprehensive literature search was undertaken to identify studies reporting outcomes of PP implantation in the setting of RIP. The primary search was conducted in PubMed/MEDLINE, supplemented by screening of grey literature (scientific meeting abstracts and Google Scholar), covering publications from January 1970 to January 2025. Reference lists of included studies were also manually reviewed to identify additional eligible publications.

Both Medical Subject Headings (MeSH) and free-text keywords were applied. MeSH terms included “priapism” and “penile Prosthesis,” and their synonyms. These were combined with free-text terms: “ischaemic priapism,” “ischemic priapism,” “refractory priapism,” “low-flow priapism,” “penile implant,” “malleable penile prosthesis,” “inflatable penile prosthesis,” “prosthesis insertion,” and “prosthesis placement.” Synonyms were pooled using the ‘OR’ Boolean operator, and priapism- and prosthesis-related search blocks were combined using the ‘AND’ Boolean operator.

### Eligibility criteria

Studies were considered eligible if they: [1] reported original clinical data on PP implantation for RIP, [2] provided outcomes related to perioperative complications, device survival, revision rates, or patient-reported satisfaction, [3] were published in English, and [4] involved human participants. Exclusion criteria were case reports with fewer than three patients; review articles, editorials, or abstracts without extractable outcome data; studies in which priapism aetiology was unclear or non-ischaemic; and reports limited to technical feasibility without complication, functional, or satisfaction endpoints.

The search strategy yielded 178 records after removal of duplicates. Two independent reviewers (RC and DT) screened all titles and abstracts identified through the search strategy. Full-text articles were then evaluated against the inclusion and exclusion criteria. Disagreements were resolved by discussion, with arbitration by senior authors (WL and DR) when necessary. Data were extracted into structured tables and cross-checked for accuracy. Following application of the inclusion and exclusion criteria, 12 studies were included in the final review.

### Limitations

Several limitations of this review must be acknowledged. First, the available evidence is largely derived from retrospective case series and institutional reports, with considerable heterogeneity in patient populations, priapism aetiology, prosthesis type, and surgical technique. This variability limits direct comparability and introduces potential bias. Second, the absence of randomized controlled trials precludes definitive conclusions regarding the superiority of early versus delayed implantation. Third, outcome measures were inconsistently reported across studies, restricting the feasibility of pooled quantitative synthesis. Fourth, publication bias may favour positive findings, while the predominance of data from high-volume tertiary referral centres reduces the generalisability of these results to wider clinical practice. Finally, although a comprehensive search strategy was employed, restriction to English-language publications may have resulted in omission of relevant studies.

## Timing of implantation

Timing of PP implantation after IP is broadly categorised into early and delayed. There is no exact definition for what constitutes “early” however it is universally understood as the timeframe that avoids implantation into fibrotic corpora.

We adopted a < 48-hour threshold to define “early” PP implantation, based on irreversible histopathological and pathophysiological changes such as sinusoidal thrombosis, cellular necrosis, and CSM necrosis and fibrosis [8, 12]. The <48-hour cutoff best reflects the transition from potentially reversible to irreversible corporal damage.

Surgical challenges under these conditions are well documented. Maximal corporal exposure, counter incisions, cavernotomes and other excavating techniques are often required to permit a narrow-based implant [[18], Table 2]. Intra-operative complications occurred in 19% of delayed cases [19–25], compared to 1% of early PPs [19–22, 26–30]. Corporal perforation and urethral injury during dilatation in the delayed group was 10% [21, 22] and 7% [21–25] respectively, compared with 1% [21, 28] reported proximal perforation in the early cohort. These differences highlight the markedly easier dilatation and implantation when surgery is performed early. Subsequently, post-operative complication and revision rate were double that of the early cases: 23% [19–25] vs 11% [19–22, 26–30], and 24% [20, 22, 24] vs 10% [20, 21, 26, 27, 29] respectively.

Device infections occurred in 5% of early cases [20, 21, 27, 29]. While not unexpected given the additive risk of ischaemia and instrumentation, it is significantly higher than rates in “virgin” implants (1%). However, this rate is higher when inserted into fibrotic corpora (8%) [20, 22]. Another article reported that 30% of delayed implants became infected with half of them needing replacement within one year [31]. Similarly, from the largest comparative study between early and delayed insertion, infection rates were 7 and 19% respectively [20]. The compiled infection rate may be lower due to improved anti-microbial practices.

Despite most men receiving a distal shunt prior to early insertion, the rate of erosion was still less than delayed implants [4% [20, 21, 27, 29] vs 11% [20, 22, 24]], where tunical integrity is compromised by difficult dissection and dilatation. One study reported erosion in 42% of delayed implants with 50% extruding through the corporotomy site, 33% migrating proximally and 17% eroding distally [22]. The authors note that all these cases received full-sized inflatable cylinders and extrusion through any weak area may occur if there is inadequate space created within the densely fibrosed corpora [22]. Comparatively, in a small study of 4 men with RIP without prior shunting, inflatable cylinder over-sizing by 0.5-1 cm was performed without complication; apart from reduced penile sensation that self-resolved at 3 months [19].

Delayed PP insertion after RIP is a technically challenging procedure with a higher rate of complication/revision leading to lower patient satisfaction [60% [20] vs 94% [19–21, 26, 27, 30]] and more complaints of penile shortening [36% [20, 25] vs 2% [20, 27]]. There is sufficient evidence to recommend early rather than delayed insertion and this is echoed by international guidelines [15–17]. On the other hand, the risk of early insertion is by no means negligible and best practices for early implantation are less clear-cut.

## Reducing risks in early implantation

### Pre-operative optimisation

The specific pre-implant risk profile of each case should guide what measures are undertaken prior to early insertion. Here, the deferral of “early” implantation is balanced against the progression to corporal fibrosis. While the onset of clinically significant fibrosis is unknown, observational accounts suggest dilatation is still relatively simple within 3 weeks of IP onset [1, 8, 20]. Deferral within this timeframe allows medical risk minimisation and provides time for appropriate referral and counselling. Infective risks are improved with time, oedema resolution, antibiotics, and medical optimisation. Allowing time for corporal defects to heal following shunting may reduce the risk of subsequent erosion. A vacuum erection device can also be used in this window to augment corporal blood flow, avoid fibrosis, and maintain length [32].

### Intra-operative measures and device selection

Both malleable penile prosthesis (MPP) and inflatable penile prosthesis (IPP) can be effectively used for early implantation in RIP. Detumescence is achieved while simultaneously providing early resumption of sexual activity and prevention of complications such as penile shortening and deformity. There are no randomised control trials comparing the two types and trends in the existing literature are by no means definitive.

MPP also acts as a static tissue expander and effectively prevents penile morphological changes due to progressive tunical and cavernous fibrosis. While this is theoretically the case for IPP, there were 3 cases [9% [20, 26, 27]] of de-novo deformity IPP was selected. All these men had failed to follow-up appropriately, resulting in delayed activation of the device at 6 weeks – 3 months. Fibrotic cicatrisation around the deflated cylinder was managed with regular device cycling and modelling in 2 of the 3 cases. Cases that used full-sized / over-sized cylinders and employed post-operative maximal inflation protocols did not report this issue [19, 22]. Thus, patient selection, pre-implant counselling and post-implant rehabilitation is important for early IPP implantation. The cylinders should also be left inflated for up to 3 weeks after surgery rather than immediate deflation the following day.

Early MPP infection [6% [20, 21, 27, 29] vs 0%] and revision rates [11% [20, 21, 27, 29] vs 3% [26]] were notably higher compared to IPP [27]. This discrepancy may reflect selection bias as MPPs were used in 84% of early cases and are the preferred option in high infection-risk cases, due to lower cost, fewer components (hence lower intrinsic infection risk) and easier removal [19–21, 26–30]. Despite this, MPP appear to have a higher risk of erosion than IPP [5% [20, 21, 27, 29] vs 0%] due to their inherent fixed length and rigidity. All erosions following early MPP placement were distal (impending glandular or urethral), became infected, required device removal and occurred following distal shunt procedures. In this situation, the advantage of PP acting as a tissue expander is lost. The only complaints of penile shortening in the early cohort were those who underwent delayed re-implantation following eroded/infected MPPs. For these reasons, prior shunting should favour a period of optimisation before early implantation.

Intra-operatively, several manoeuvres have been described to mitigate the risk of erosion following distal shunts. Careful distal dilatation is facilitated by using Hegar instead of Brooks dilators, whose olive-shaped head generate less tissue resistance and are associated with higher chance of distal perforation. Dilatation up to the coronal sulcus, not into the glans, is similarly recommended. MPP cylinders should be intentionally downsized by about 1 cm and fixed to the corporotomy site to prevent distal migration, pressure necrosis and subsequent erosion. In one study, suture fixation was performed in all 12 patients (11 of whom received distal shunts) and no complications were reported during median 24-month follow-up [28]. The caveat to undersized cylinders is their propensity to glans hypermobility. The device can then be exchanged to an upsized IPP (up to 1.6 cm longer) several months after the first operation. Good patient satisfaction has been reported with this algorithm but is contingent on the ability to undergo two operations.

Where no distal shunt has been performed, limited evidence supports the safe use of full-sized malleable or inflatable cylinders [19, 30]. Appropriate optimisation strategies prior to early placement may allow safe implantation of full sized cylinders after distal shunting in men who desire a single operation; however further research in this space is required.

## Conclusion

When conservative management has failed and IP duration > 48 h, early PP implantation should be considered. While early insertion has a significantly reduced risk of complications compared to delayed implantation, risk mitigation strategies should be employed on a case-by-case basis and patient counselling is paramount. Slight deferral up to 3 weeks from IP onset is acceptable and likely favourable where distal shunt procedures have been performed. Glans preserving shunt procedures should be considered as first line, however more research is required to demonstrate its superiority in erectile function preservation, not only detumescence.

Future directions should focus on standardizing surgical protocols and timelines, reducing heterogeneity in management to better compare outcomes.
